# Breathing Re-Education and Phenotypes of Sleep Apnea: A Review

**DOI:** 10.3390/jcm10030471

**Published:** 2021-01-26

**Authors:** Patrick McKeown, Carlos O’Connor-Reina, Guillermo Plaza

**Affiliations:** 1 Buteyko Clinic International, Loughwell, Moycullen, Co., H91 H4C1 Galway, Ireland; patrick@buteykoclinic.com; 2 Otorhinolaryngology Department, Hospital Quironsalud Marbella, 29603 Marbella, Spain; carlos.oconnor@quironsalud.es; 3 Otorhinolaryngology Department, Hospital Quironsalud Campo de Gibraltar, 11379 Palmones, Spain; 4 Otorhinolaryngology Department, Hospital Universitario de Fuenlabrada, Universidad Rey Juan Carlos, 28042 Madrid, Spain; 5 Otorhinolaryngology Department, Hospital Sanitas la Zarzuela, 28023 Madrid, Spain

**Keywords:** obstructive sleep apnea, breathing re-education, dysfunctional breathing, myofunctional therapy, phenotypes

## Abstract

Four phenotypes of obstructive sleep apnea hypopnea syndrome (OSAHS) have been identified. Only one of these is anatomical. As such, anatomically based treatments for OSAHS may not fully resolve the condition. Equally, compliance and uptake of gold-standard treatments is inadequate. This has led to interest in novel therapies that provide the basis for personalized treatment protocols. This review examines each of the four phenotypes of OSAHS and explores how these could be targeted using breathing re-education from three dimensions of functional breathing: biochemical, biomechanical and resonant frequency. Breathing re-education and myofunctional therapy may be helpful for patients across all four phenotypes of OSAHS. More research is urgently needed to investigate the therapeutic benefits of restoring nasal breathing and functional breathing patterns across all three dimensions in order to provide a treatment approach that is tailored to the individual patient.

## 1. Introduction

Obstructive sleep apnea hypopnea syndrome (OSAHS) is a chronic sleep-related breathing disorder that is increasingly widespread and represents a significant cost to health [1,2]. In recent years, it has been found that OSAHS is not merely an anatomical issue, but that factors including arousal threshold, unstable breathing control and poor upper airway recruitment contribute. There is evidence to suggest that individuals who experience mixed apneas may have fundamental differences in respiratory control, and that these present as greater breathing pattern variabilities during wakefulness [3]. A bi-directional relationship exists between dysfunctional breathing during wakefulness and disordered breathing during sleep [4]. Equally, breathing during wakefulness is a strong determinant of breathing during sleep [5]. It stands to reason that if breathing is dysfunctional during the day, it will not be functional at night. Jack et al. surmised that abnormal ventilatory responses may, in fact, become part of the respiratory “make-up” of the individual [6].

Dysfunctional breathing patterns affect 9.5% of the general population [7], increasing to 30% in the asthma population and 75% in the anxiety population [8]. It is possible to manipulate breathing patterns during wakefulness using exercises that target the biochemistry, biomechanics and frequency [9,10,11]. In this way, the breath can be “trained” to restore nasal breathing, improve diaphragm function, slow the respiratory rate and increase tolerance to changes in arterial carbon dioxide (CO_2_) pressure. If poor breathing patterns during wakefulness can be addressed, it is likely that this may provide a mechanism whereby sleep-disordered breathing can also benefit.

### 1.1. Breathing Re-Education

Breathing re-education (BRE) focuses on the patient’s breathing pattern, as dysfunctional breathing, such as chronic hyperventilation, is known to contribute to hypocapnia and related physical and mental problems, e.g., asthma and anxiety or panic disorders [12]. The Buteyko breathing technique was introduced in Russia in the 1950s by Dr. Konstantin Buteyko. Buteyko identified various dysfunctional breathing habits—such as mouth breathing and upper chest breathing—as being among the major causes for chronic hyperventilation. Consequentially, he introduced breathing exercises based on breath-holding maneuvers and breath control to guide patients back to the normal nasal/diaphragmatic breathing pattern. The aim, to reduce breathing volumes and restore metabolic balance.

BRE is a therapeutic intervention based on the following fundamentals [13]:

- Establishing full-time nasal breathing during wakefulness and sleep.
- Correcting the resting posture of the tongue.
- Slowing the respiratory rate.
- Using breath-hold time (BHT) to establish chemosensitivity to CO_2_.
- Restoring diaphragm function and the lateral expansion of the lower ribs.
- Reducing the minute volume towards normal to regulate levels of CO_2_.

**Figure 1:**
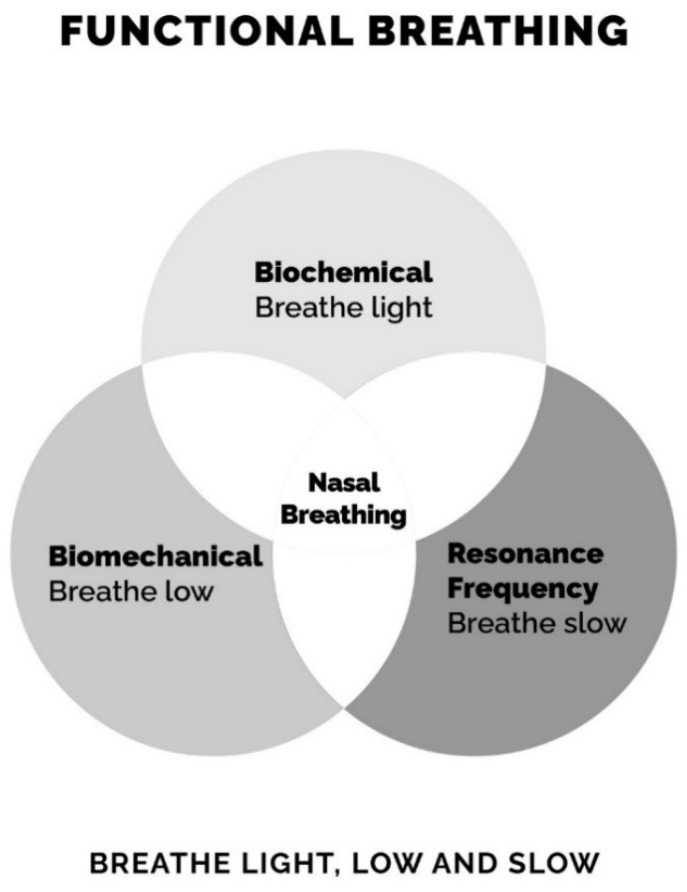
Breathing re-education is based on three dimensions. The causes of dysfunctional breathing are biomechanical, biochemical and psychological. Breathing re-education (BRE) approaches these from three dimensions, each underpinned by full-time nasal breathing. (1) Biomechanical, breathe low to engage the diaphragm. (2) Biochemical, breathe light, reduce tidal volume and lessen chemosensitivity to CO_2_. (3) Resonant frequency—slow breathing at six breaths per minute.

**Figure 2:**
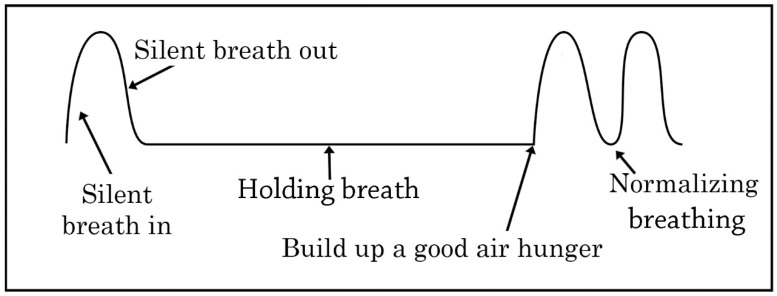
Breathing re-education. A video demonstration of decongesting nose exercise is available as Supplementary Materials (Video S1).

The key to treating dysfunctional breathing lies in viewing breathing pattern disorders from a three-dimensional perspective (Figure 1). Dysfunctional breathing can be triggered by biomechanical, biochemical or psychological factors. As such, it can be treated from a biomechanical or biochemical dimension or using cadence/coherent breathing (Figure 2). Cadence/coherence is the practice of slowing the breathing rate to six breaths per minute (bpm), a respiratory rate proven to optimize parameters, including heart rate variability, respiratory sinus arrhythmia, baroreflex function and blood gas exchange [14], to reduce dead space in the lungs [15] and to improve sympathovagal balance [15].

To decongest the nose, instruct the student to perform the following:

- Take a normal breath in and out through your nose;
- Pinch your nose with your fingers to hold your breath;
- As you hold your breath, move your body or gently nod your head up and down;
- Hold your breath for as long as you can—until you feel a strong air hunger;
- Let go of your nose and breathe through it as calmly as possible.

Repeat 6 times with a 30–60 s rest between each repetition.

BRE is of considerable interest to people with asthma and is recommended in evidence-based guidelines as possible adjuvant treatment for patients whose symptoms are not adequately controlled by pharmacological treatment. The current evidence base for BRE in asthma has been assessed as convincing by some systematic reviews, and the most recent Cochrane review has reported encouraging trends [16]. The BREATHE (Breathing Retraining: A Trial of Home Exercise) randomized controlled trial (RCT) in 655 primary care patients following self-guided breathing retraining showed that BRE was an effective and cost-effective way to improve quality of life for adults with asthma [17]. A digital intervention research group subsequently offered free online access to the intervention for people with asthma and healthcare professionals with excellent results [18]. A very recent RCT has shown the Buteyko breathing technique effective in children with asthma [19].

The purpose of this review is to evaluate the possible application of BRE to OSAHS, and the effect that BRE might have in the four phenotypes that are currently described in OSAHS patients.

### 1.2. Prevalence of OSAHS

Sleep-disordered breathing is a widespread condition with significant public health outcomes [20] and it is becoming ever more prevalent [21]. Its incidence also increases with age [22]. In the 30–49-year age group, OSAHS is present in 9% of women and 26% of men. In the 50–70-year age group, it affects 27% of women and 43% of men [23]. While these figures are alarming, research suggests that the majority of people with OSAHS still remain undiagnosed and untreated [4,5,6,7,8,9,10,11,12,13,14,15,16,17,18,19,20,21,22,23,24,25,26].

There is a lack of enthusiasm for existing treatment options, and this contributes to poor treatment uptake. The gold standard treatment for OSAHS is continuous positive airway pressure (CPAP). but many factors can play a role in non-adherence. Claustrophobia, nasal obstruction and poor social support can all negatively impact CPAP use [27]. Mouth leaks are a common problem, potentially contributing to arousals and drying the mucosa in the airways [28]. Chin straps are used to counter this issue, but there are limited data to indicate their efficacy [28]. In some instances, patients refuse treatment due to fears that a diagnosis will prompt the withdrawal of their driving license [23]. Sleep deprivation contributes to around 109,000 road traffic collisions resulting in injury and 6400 fatal traffic accidents annually in the US [29], and laws for drivers prohibit patients with uncontrolled OSAHS from driving.

In recent years, there has been an uptake in the use of mandibular advancement devices (MAD) in treating OSAHS [30]. MADs prevent airway collapse by protruding the mandible to alter the position of the tongue and jaw. These devices can cause side effects, including excessive salivation, dry mouth, dental pain, gingival irritation, myofascial pain and temporomandibular joint pain [31,32,33]. MADs have better patient adherence than CPAP but are not recommended for patients with severe OSAHS.

A further concern is that primary care physicians may fail to sufficiently explore the avenue of early OSAHS diagnosis, especially if the patient does not present with daytime fatigue and a high body mass index [19]. As many as half of all people with OSAHS are not obese and 25% of those with moderate OSAHS demonstrate neither subjective nor objective sleepiness [19]. It is, therefore, essential to perform sleep studies in order to correctly diagnose the disease.

### 1.3. The Four Phenotypes/Endotypes of OSAHS

The field of sleep medicine has changed radically in the last seven years with the recognition that OSAHS is not simply an anatomical issue. Upper airway collapsibility and craniofacial anatomy remain fundamentally important in the development of OSAHS [34]. However, the cause of OSAHS differs from one individual to another. Three non-anatomical phenotypes have now been identified, indicating that OSAHS can develop due to multiple contributing factors. It is likely that the combination of these factors varies significantly between patients [19].

**Figure 3:**
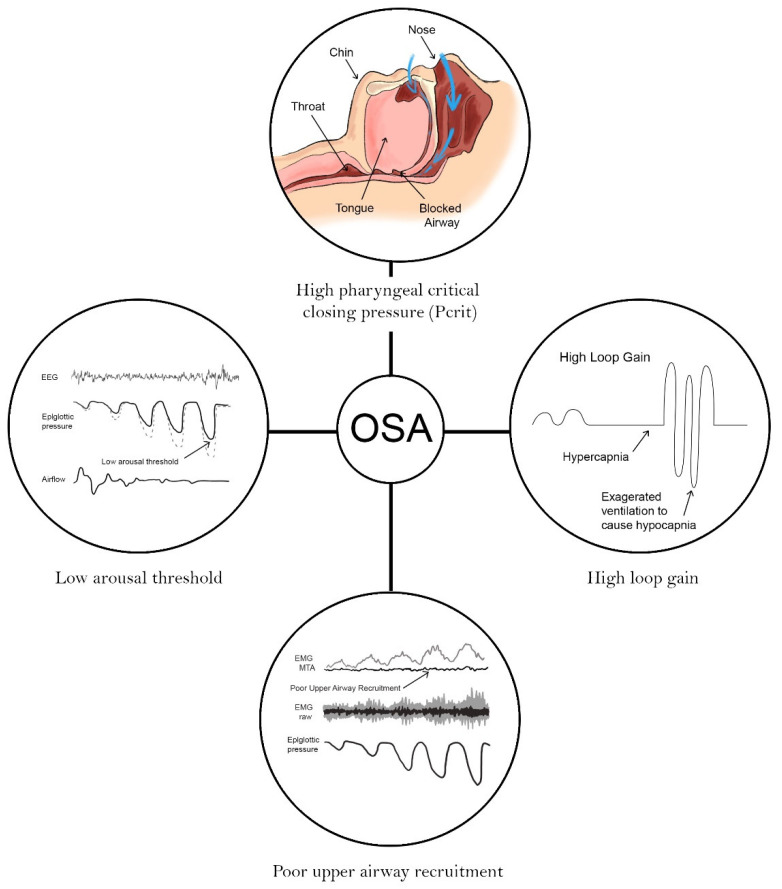
Four phenotypes/endotypes in obstructive sleep apnea hypopnea syndrome (OSAHS), modified from Eckert et al. [34,35].

The four phenotypes, as defined in 2013 research by Eckert et al., are pharyngeal critical closing pressure (Pcrit), loop gain, upper airway recruitment and arousal threshold [34]. This concept has evolved further into one of four endotypes underlying the four phenotypes [35,36,37,38,39,40]; a development that facilitates a model of personalized treatment approaches for individual OSAHS patients (Figure 3) [36,37].

Treatment outcomes for the patient are strongly influenced by whether or not treatment is tailored to the phenotypes of the individual. For instance, a patient with high loop gain will not respond favorably to MAD [5]. The importance of this should not be underestimated. Eckert demonstrated that pathophysiological traits varied substantially between patients with the same condition. Of those patients with OSAHS, 36% had minimal genioglossus muscle responsiveness during sleep, 37% showed a low arousal threshold, 36% had high loop gain and 26% demonstrated compound nonanatomic features [34]. Craniofacial structure and pharyngeal anatomy play an important role. Overall, the upper airway is more collapsible in patients with OSAHS. However, Eckert found that 19% of his OSAHS subjects had a comparatively non-collapsible upper airway, similar to many of the controls. In these patients, loop gain was almost two times higher than it was in patients with a highly collapsible airway [34,35].

Phenotyping of OSAHS will have an important use for the sleep specialist, promoting the development of precision medicine and personalized management. A correct concept of phenotypes and endotypes of OSAHS is thus needed to understand the role that BRE should play in the multidisciplinary management of this disease [35,36,37,38,39,40].

#### 1.3.1. Pharyngeal Critical Closing Pressure (Pcrit)

Pcrit is the gold standard of OSAHS assessment in terms of functional anatomy. It is used to measure the collapsibility of the airway in sleep-disordered breathing conditions from snoring to OSAHS [19,41]. Pcrit is defined by the level of negative suction pressure required to close the airway during sleep. This can be impacted by airway narrowing, and by airway collapsibility due to impaired function of upper airway dilator muscles. A narrow airway creates greater resistance and is, therefore, more vulnerable to collapse [42]. Equally, airflow must be taken into account. When breathing is hard and fast, the flow of air increases. This adds to the negative suction pressure present in the airway and, therefore, increases the likelihood of airway collapse. A patient is considered to have a high Pcrit when the airway collapses easily. While the apnea/hypopnea itself is characterized by a drop in airflow, apneic events are commonly preceded by excess airflow. This is why apneas self-perpetuate when the patient resumes breathing with a large gasp of air.

Factors that contribute to high Pcrit include deposits of fat around the pharynx and torso. Abdominal obesity compresses the abdomen and thoracic cavities causing an anatomical reduction in lung volume. This reduces tracheal tension and thus impairs the function of the upper airway dilator muscles. The same effect occurs during rostral fluid shift in patients with congestive heart failure. Rostral fluid shift involves fluid that has collected in the legs during the day migrating to the neck during sleep, restricting the function of the upper airway [43]. Abdominal fat also compromises the function of the diaphragm, reducing the amplitude of diaphragm movement. It is known that a reduction in diaphragm amplitude reduces lung volume [44], and that a reduced lung volume leads to greater collapsibility of the throat.

#### 1.3.2. Loop Gain

Loop gain is a measure of the stability of ventilatory chemoreflex control. In other words, it reflects chemosensitivity to CO_2_. Patients with high loop gain have an exaggerated response to minimal changes in CO_2_. Messineo et al. assessed loop gain using breath holding and found that high loop gain is directly related to low breath-hold time (BHT) [5]. If loop gain represents respiratory chemosensitivity, it can be reasonably extrapolated that exercises to decrease respiratory chemosensitivity will help patients with high loop gain.

When the breathing stops during an apnea, CO_2_ is unable to leave the body via the lungs and so builds up in the bloodstream. The respiratory process is controlled by the levels of oxygen, CO_2_ and hydrogen ions in the arterial blood. Of the three, CO_2_ provides the most significant ventilatory stimulus. Rassovsky et al. state that an increase in pCO_2_ of just 2–5 mmHg can increase ventilation more than twofold [45].

When breathing resumes after an apnea, individuals with high loop gain demonstrate exaggerated ventilation in response to minimal increases in carbon dioxide [46]. A fast respiratory rate and high tidal volume cause the depletion of CO_2_ and the switch from hypercapnia to hypocapnia. When the level of blood CO_2_ is too low, the brain is unable to send appropriate signals to breathe, and this can result in a central apnea [47]. Buterbaugh et al. demonstrate decreased cerebrovascular reactivity in response to breathe holding [48]. At the same time, when the respiratory signals are inhibited, the respiratory muscles designed to open the airway become less effective. Jordan et al. state that the activity of these airway dilator muscles alters so that when central respiratory drive is low, upper airway dilator muscle activity is also low. This creates high levels of resistance in the airway and increases the risk of airway collapse [49].

High loop gain can lead to a vicious cycle in which breathing resumes with such exaggerated force that the respiratory signals are inhibited. This can cause a central apnea to occur. At the same time, increased collapsibility of the throat produces an obstructive apnea. For this reason, high loop gain contributes to perpetuating apneas. This is supported by evidence that loop gain predicts apnea-hypopnea index (AHI) scores [44].

Messineo et al. tested 20 patients in an overnight study using breath-hold time during wakefulness to determine the loop gain during sleep. The study tested maximal breath-hold duration and ventilatory response in the first two breaths following a 20 s breath hold—a duration that it was expected all participants could tolerate. Higher loop gain during sleep correlated with both a shorter maximal breath-hold time and a larger ventilatory response to a 20 s breath hold during wakefulness [5].

#### 1.3.3. Upper Airway Recruitment

The human pharynx is unique in that it lacks rigid, bony support [19]. Depending on the dynamic balance that exists between negative suction pressure within the airway and neural drive to the upper airway dilator muscles, the pharynx is susceptible to collapse during sleep [19]. Eckert et al. point out that the ability to translate upper airway neural drive to the mechanical contraction of upper airway muscles may be compromised in some patients with OSAS, suggesting that the mechanical efficacy of upper airway contraction plays a potential role in the recruitment of upper airway dilator muscles [34].

Osman et al. state that there are more than 20 muscles in the upper airway. These are involved in both respiratory and non-respiratory functions, including breathing, chewing, speech and swallowing. In healthy people, activation of the upper airway muscles effectively opposes the negative suction pressure created during inhalation. This is also the case in patients with OSAHS during wakefulness. However, during sleep, reduced activity of these dilator muscles combined with a narrow airway can prompt airway collapse [19]. Upper airway recruitment threshold is defined by the level of stimulus required to activate the upper airway dilator muscles. A poor muscle responsiveness to upper airway collapse during sleep—low upper airway recruitment threshold—may increase the severity of OSAHS [50,51]. Threshold stimulus intensity for the laryngeal adductor reflex is significantly higher in OSAHS subjects [52]. Furthermore, it has been demonstrated in comparison testing of a two-point “palatal discrimination response” that OSAHS subjects have significantly higher dysfunction in palatal sensory input than non-OSAHS subjects [53].

#### 1.3.4. Arousal Threshold

In the simplest of terms, arousal threshold refers to whether the patient is a light sleeper or a deep sleeper. The propensity to wake frequently from sleep correlates with insomnia, another sleep disorder that is commonly linked to autonomic imbalance [54]. Insomnia is known to increase the risk for incidence and severity of depression, depressive episodes and suicide, and studies have demonstrated that OSAHS can also contribute to the pathology of depression [55]. When insomnia and OSAHS presented comorbidly in the same patient, depression scores were higher than in insomnia patients without OSAHS. Grandner and Malhotra speculate that the mechanism by which OSAHS and insomnia may add to the severity of depression is the arousal threshold. A key characteristic of insomnia is cortical hyperarousal which “likely results in a decreased arousal threshold” [54].

Arousal threshold is defined by the level of intra-esophageal pressure and the amount of change in the concentration in arterial CO_2_ required to trigger arousal [44]. Patients with a low arousal threshold and poor upper airway recruitment will wake before the dilator muscles have activated to open the airways, meaning they experience frequent, unnecessary arousals. This kind of light sleep is problematic because continuous arousals lead to sleep fragmentation, fatigue and poor daytime function. It has also been proven that individuals with the greatest risk of all-cause mortality are those with a low arousal threshold [56]. Butler et al. found that short duration of respiratory events, which is indicative of a low arousal threshold, predicts mortality in both men and women [56].

If the upper airway dilator muscles are not functioning properly, sleep that is too deep can also present a problem. If the arousal threshold is so high that the patient fails to arouse during an apnea, the breathing can stop for a long time, leading to greater oxygen desaturation. High arousal threshold has, for instance, been implicated in sudden infant death syndrome [57].

### 1.4. Sex Differences in OSAS Prevalence

Breathing pattern disorders and chronic hyperventilation are more prevalent in women than men. This may be due to hormonal influences. Progesterone stimulates the respiratory rate in the luteal phase of the menstrual cycle (the phase after ovulation and prior to menstruation). During this time, levels of CO_2_ can drop by up to 25%. Stress can further increase hyperventilation when CO_2_ is already low [58].

Women have a later onset of OSAHS, and AHI severity did not increase until age 50 years and older. These gender differences decreased with age. Studies have suggested that hormonal changes experienced by women during menopause may be responsible for an increased AHI in women of menopausal age [15,59,60].

It has been proven that OSAHS and sleep-disordered breathing (SDB) increase in postmenopausal women. LoMauro and Aliverti suggest that sex hormones play a protective role in airway health in women [61]. The literature in this area is sparse, but Ott et al. found significant correlations between symptoms of pre-menstrual syndrome and hyperventilation [62]. Gargaglioni et al. reported that while OSAHS is more prevalent in men, the incidence of OSAHS in women increases 200% once menstruation ceases [63]. Pre-menopausal women with severe OSAHS have a much lower progesterone concentration than healthy women in the same demographic and pre-menopausal women with mild OSAHS. Stavaras et al. suggest that the menopausal state itself plays a part in OSAHS phenotypes [64], as indicated by the fact that gender differences in the prevalence of OSAHS decrease in postmenopausal women.

Changes in body-fat distribution are likely to contribute to OSAHS in postmenopausal women [65]. After menopause, women tend to have more fat on the tongue, neck and abdomen. Fat in these areas is a common anatomical factor in sleep apnea, contributing to Pcrit. However, excess weight affects men and women differently in terms of respiration [66,67]. Kunitomo et al. examined the incidence of SDB in obese men and women and reported that obese women had a heightened chemosensitivity to hypoxia and hypercapnia compared to women of a healthy weight. The same was not the case in men. This suggests a greater vulnerability to high loop gain in women who are overweight [68].

## 2. Applying the Three Dimensions of Breathing Re-Education to Each of the Four Phenotypes of OSAHS

OSAHS is caused by the interaction of several key traits of upper airway anatomy and neuromuscular control [35]. These contribute to the condition in varying degrees from one individual to another. Current treatment options each primarily targeted a single phenotype of OSAHS. It seems important, therefore, to examine novel treatment opportunities so that treatments can be personalized depending on which phenotypes present, ensuring successful treatment outcomes.

There is currently limited research into the relationship between breathing pattern disorders and the phenotypes of sleep apnea. Equally, the application of BRE for OSAHS has not been studied. It is known, however, that mouth breathing during sleep increases the severity of OSAHS [69], and techniques integral to BRE correlate with concepts directly relevant to the various phenotypes. Approaches involving BRE that have been investigated for OSAHS all incorporate some type of breathing modulation and/or control. Methods have included wind instrument playing, orofacial myofunctional therapy and didgeridoo playing (which is known to strengthen the pharyngeal muscles) [70,71], singing exercises, respiratory muscle strengthening exercises, diaphragmatic breathing pattern training and the Buteyko Method [4].

In a review of 14 articles, Courtney describes an interest in treatments that address the four phenotypes of OSAHS, emphasizing the bi-directional relationship between breathing during the day and breathing at night [4]. This relationship exists in people with panic disorder and severe daytime dysfunctional breathing. There is also evidence that some OSAHS patients who have high chemosensitivity to CO_2_ and high loop gain during sleep maintain these characteristics during wakefulness [5] (Table 1).

### 2.1. Breathing Re-Education and Pcrit

The foundation of BRE includes switching from oral breathing to nasal breathing during rest, exercise and sleep. Oral and oro-nasal breathing is common in sleep apnea and increases with age. Computational fluid dynamics results during nasal and oral breathing revealed that oral breathing is the primary condition leading to pharyngeal airway collapse based on the concept of the Starling Resistor model [78]. Once an individual reaches the age of 40 years, he or she is six times more likely to spend at least 50% of sleep time breathing through an open mouth [79]. In a recent study of 65 males and 30 females with established OSAHS [69], 36.8% breathed nasally during sleep, 11.6% had oral breathing, and 51.6% had oro-nasal breathing.

The anatomical size of the airway is influenced by whether the mouth is open or closed. During nasal breathing, it is possible for the tongue to rest in the roof of the mouth [80]. In this position, the tongue is less likely to encroach on the airway. Mouth breathing is typically thoracic rather than diaphragmatic [73,74]. Yi et al. used fluoroscopy to analyze diaphragm excursion in children who breathed nasally and those who breathed orally. Diaphragm amplitude was less in children who mouth breathed. The researchers also found that when significant nasal obstruction is present, as it is during mouth breathing, there is a conscious effort to overcome the obstruction to breathing involving increasing inspiratory effort by means of the accessory muscles [74].

When the diaphragm is not properly engaged, diaphragmatic excursion is less [81]. When the amplitude of diaphragm movement is compromised, there is a subsequent reduction in lung volume [82]. When lung volume decreases, the throat collapses more easily. In this way, mouth breathing causes a reduction in lung volume and increases the collapsibility of the throat [49].

Nose breathing has been shown to produce greater amplitudes of diaphragm movement and increase lung volume [83,84]. The consequent increase in functional residual capacity (the volume of air that remains in the lungs after a passive exhalation) is believed to improve gas exchange and, therefore, the pressure of arterial oxygen [85].

It has also been suggested that intensive practice of diaphragm breathing exercises prevents the collapse of the airway by improving the strength of the entire respiratory tract and enhancing the ability of the central nervous system to organize breathing [86].

Conversely, mouth breathing is linked with greater severity of OSAHS. Fitzpatrick et al. examined healthy subjects to compare nasal and oral breathing routes. The study found that when breathing was through the mouth, upper airway resistance during sleep was 2.5 times greater than when breathing was through the nose [72]. Hsu et al. reported that mouth breathing was strongly associated with greater oxygen desaturation and more significant upper airway collapse. The AHI during mouth breathing was 52.15. For those patients with oro-nasal breathing it was 42.09, and for those who breathed nasally it was 27.40 [69].

This finding is in line with the results of 1997 research by Young et al., which used data from subjective questionnaires and objective in-laboratory measurements to examine history of nasal congestion and sleep problems. Those participants who reported nasal congestion due to allergy were 1.8 times more prone to moderate or severe SDB than those patients with no nasal congestion due to allergy [87].

Furthermore, it has been demonstrated that mouth breathing is a cause of CPAP non-compliance [88]. It has also been found in research investigating the treatment of the nose using intranasal steroids, that chronic nasal obstruction plays a minor role in SDB. These studies failed to establish whether or not participants were breathing through the nose. In not one of nine trials involving external nasal dilators, topically applied nasal steroids, nasal decongestant and surgical treatments [89] did the researchers ask whether, having had a procedure to open up the nose, the patients were actually using the nose to breathe during wakefulness and sleep. It is vital that the post-surgical follow-up for Ear Nose and Throat (ENT) patients and children undergoing adenotonsillectomy should include a period of breathing rehabilitation. Typically, when patients undergo turbinate reduction surgery, surgery for a deviated septum or removal of the adenoids, mouth breathing will continue in most cases due to the patient’s existing mouth breathing habit [90].

In children, persistence of mouth breathing post tonsillectomy and adenoidectomy plays a role in the worsening of the AHI, frequently within three years [91]. The fact that perceived nasal obstruction does not preclude the ability to breathe nasally was demonstrated by Zaghi et al., who reported that 80% of 633 mouth-breathing study participants, including 315 children aged 3–11 years, were able to comfortably breathe through the nose for at least three minutes when their lips were taped [92].

Mouth breathing is an important factor, especially in older patients. It is well known that mouth breathing contributes to snoring as well as apneas and hypopneas [93]. Mouth breathing is also associated with a compromised response to hypoxia of the genioglossus [94]—the primary muscle responsible for protruding the tongue.

Our own empirical evidence indicates that the only way to ensure nasal breathing during sleep is to use supports such as paper tape across the lips, chin up strips or MyoTape^®^ (elasticated cotton tape designed to surround the mouth). There is an argument for taping the mouth during sleep regardless of whether breathing is through the mouth, nose or oro-nasally. Meurice et al. found that just opening the mouth increases the collapsibility of the upper airway independently of any nasal obstruction and without changes in the breathing route [95]. This is thought to be due to mechanical obstruction of the upper airways caused by a combination of upper airway narrowing, and a reduction in the efficiency of contraction in the upper airway dilator muscles. The increase in collapsibility was not large enough to be of clinical significance in individuals with normal airway collapsibility, but in patients with OSAHS, the changes could have significant clinical implications [91].

### 2.2. Breathing Re-Education and Loop Gain

Chemosensitivity to CO_2_ can be estimated by measuring breath-hold time (BHT) [5,96]. One of the fundamentals of BRE is the use of a breath hold on exhalation as an objective measure of breathlessness. It is known that high loop gain during sleep is determined by a low breath-hold time during wakefulness [5]. Short breath holding time is a known trait of individuals with chronic idiopathic hyperventilation and other types of dysfunctional breathing [97,98,99,100,101,102,103].

Keisel et al. [102] proposed a test consisting of four questions from the Functional Movement Screen (FMS™) and a BHT of 25 s and confirmed that dysfunctional breathing can be predicted by the patient’s ability to hold the breath for 25 s. The important thing to note is that by using breathing exercises that reduce the respiratory rate to lower minute ventilation for periods of time during rest it is possible to improve BHT, and to reduce chemosensitivity to CO_2_. Since chemosensitivity to CO_2_ and BHT are both predictors of loop gain, BRE may reduce the loop gain by increasing BHT and lowering chemosensitivity to CO_2_.

It is apparent that treatment of loop gain is important in the treatment of OSAHS, especially in patients who do not respond to MAD and surgery [5,104]. MADs do nothing to decrease loop gain [102] and MADs are less effective when a high loop gain is present [105]. Loop gain is not affected by CPAP, although CPAP is better tolerated in those subjects with high loop gain [106].

### 2.3. Breathing Re-Education, Myofunctional Therapy and Upper Airway Recruitment

Nasal breathing harnesses the gas nitric oxide, which plays a role in the maintenance of muscle tone and regulation of neuromuscular pathways in the pharyngeal muscles [13]. Individuals with OSAHS tend to have minimal or poorly coordinated upper airway muscle dilation during inhalation [95,96]. The upper airway muscles and breathing are “neurologically and functionally linked” [107]. Brown found that subjects with the highest AHI “typically had little movement of the tissues surrounding their airway during wakefulness” [108].

It has been found that individuals with OSAHS have reduced respiratory muscle strength compared with individuals of the same age and gender without OSAHS [4]. According to Courtney, this may be of clinical significance “given that the magnitude and stability of respiratory motor output” to the muscles of the upper airway and chest wall are “major contributors to all types of sleep apnea” [4].

BRE includes exercises to improve the strength and function of the inspiratory muscles, in particular, the diaphragm. Because of the small size of the nostrils relative to the mouth, breathing through the nose during wakefulness imparts a resistance to airflow that is at least 50% greater than the resistance from mouth breathing [109]. It may appear that lower resistance might be a positive thing, but the increased pressure in the lungs during nasal exhalation causes the air to be denser, simulating a lower altitude where the partial pressure of oxygen in the air is higher. This improves perfusion into the alveoli [109]. The healthy diaphragm amplitude associated with nasal breathing improves venous return to the heart [14], reducing cardiac effort [109]. Breathing through the nose during wakefulness may also help to improve and maintain diaphragm strength [73,81].

The resting posture of the tongue is relevant in OSAHS. The genioglossus muscle in the tongue plays a key role in maintaining an adequate airway [110,111]. Individuals who mouth-breathe have habitually poor tongue posture and increased likelihood of the tongue falling back into the airway. For these patients, it may be beneficial both to re-educate the tongue muscles and to improve the tone and function of the upper airways [112]. Myofunctional Therapy (MT) exercises may increase tone in the oral and/or oropharyngeal muscles and even reduce the amount of fat deposited on the tongue [110]. This indicates that there may be a place for MT in the treatment of OSAHS. However, MT is very demanding, as demonstrated by the important percentage of dropouts in studies that are commonly cited to illustrate its efficacy [111,113]. Therefore, the strategy of BRE should not be to add on MT maneuvers but to refine which components are most effective in order to facilitate acceptance and long-term adherence to such programs.

Wishney et al. state that MT offers a potential way to increase tone in the upper airway muscles and restore nasal breathing, and that it can provide a successful adjunct therapy for OSAHS in adults and children [114]. The debate surrounding MT remains unresolved due to a lack of quality research. At present, there is insufficient evidence to recommend MT as a one-size-fits-all treatment for OSAHS, but there are data to suggest that it can be effective in improving the function of the upper airway dilator muscles and resting tongue position.

In a review of the relevant literature, Camacho et al. concluded that MT yields a reduction in AHI of around 50% in adults and 62% in children [113]. The review reveals that studies with control groups report little or no improvement in AHI for controls compared with improvements in participants treated with MT. The authors also clearly demonstrate improvements in lowest oxygen saturation of between 3 and 4%, with data from a number of independent studies recording a mean difference in SPO_2_ before and after MT of 4.19% [113]. The manuscripts included in this review do not share a single methodology or group of exercises, but outcomes were considered consistent.

Therefore, more research is needed to identify the pathophysiology and mechanisms whereby MT is effective for some patients with OSAHS. The review recommends that future studies utilize the standardized exercises that have been developed and used by Guimaraes et al. [115], who have the most experience with the therapy. As with BRE, MT is based on an integrative approach involving several exercises, and so it is not possible to define which of the exercises contribute most significantly to treatment outcomes. The review concludes that lowest SPO_2_, sleepiness and snoring all improved in adults as a result of MT and that the therapy could provide a useful adjunct to other forms of OSAHS treatment [115].

The main problem with MT is the proper selection of the patient to the therapy. O’Connor et al. suggest that patients with an absence of nasal obstruction, no restriction in tongue movement and low airway muscle tone (as diagnosed using the Iowa Oral Performance Instrument (IOPI)) are the best candidates for MT [116]. It is also important to improve long-term adherence to MT. For this purpose, smartphone apps such as AirwayGym^®^ have been developed [117]. In a randomized trial with severe OSAHS patients, the AHI decreased by 53.4% from 44.7 (33.8–55.6) to 20.88 (14.02–27.7) events/hour (*p* < 0.001). The tongue pressure increased from 39.83 (35.32–45.2) to 59.06 (54.74–64.00) kPa (*p* < 0.001). The AHI correlated significantly with the tongue pressure. There was an adherence rate of 90% in the intervention group [118].

Suzuki et al. [119] recently ran a longitudinal study of 32 patients undergoing 6 months of MT. AHI decreased significantly from 34.7 to 29.0/h (*p* = 0.03), while tongue pressure significantly increased from 35.9 to 45.6 kPa (*p* < 0.01). Seven patients (22%), including six of the 12 patients with moderate OSAHS (50%), experienced successful CPAP discontinuation.

In 2019, Huang et al. published the first study to indicate that MT can restore nasal breathing during sleep [120]. All-night nasal breathing is the only marker of successful upper airway treatment. In a 6-month follow-up of children who had undergone surgery to remove the tonsils and adenoids, it was found that those with good MT compliance breathed nasally during sleep.

Diaféria et al. [121] studied 100 men with a mean age of 48.1 years, BMI of 27.4, Epworth sleepiness scale (ESS) score of 12.7 and an AHI of 30.9. The men were divided into three groups and treated using MT, CPAP or combined MT and CPAP. All participants showed a decrease in ESS and snoring, but these improvements were maintained in the MT group after the “washout period”, whereas readings returned to pre-treatment levels in the CPAP and combined groups. AHI was reduced in all the patients. The MT and combined groups demonstrated improved soft palate and tongue muscle strength. Where MT was offered in conjunction with CPAP, participants showed increased CPAP adherence compared with those patients who were using CPAP alone [118]. However, a selection bias exists in this group as the MT and combined subjects were monitored more frequently than the CPAP-only subjects, which the authors believe may have encouraged adherence.

A review from de Felício et al. showed that MT is successful in reducing snoring and OSAHS and improving quality of life in adults. It is also effective in treating children with residual apnea, and it improves CPAP compliance and adherence. Only a limited number of clinical studies currently exist into MT, and it is necessary to analyze the long-term effects of treatment to discover whether it contributes to changes in the musculature [122].

### 2.4. Breathing Re-Education and Arousal Threshold

During nose breathing, sleep is deeper. In 1991, Smith et al. identified a neural circuit within the brainstem called the preBötzinger complex (preBötC) [123]. This neural circuit was thought to be responsible for generating respiratory rhythm. In 2017, Yackle et al. found a small, molecularly defined neuronal subpopulation in the mouse preBötC, the primary breathing rhythm generator, believed to regulate the balance between calm and arousal behaviors [124]. In humans, increasing ventilation induces arousal from sleep regardless of the stimulus producing this rising drive to breathe [125]. Nose breathing creates greater resistance to airflow (10–20%) [126,127], slowing the respiratory rate. It follows that nasal and slow breathing could protect against unnecessary arousals. Promoting nasal breathing should be the first goal of all ENT specialist. Nasal breathing with mouth closed and tongue in the papilla prevents normal velopharynx to collapse. When there is a pathological velopharynx it is necessary to adopt other measures.

Low arousal threshold and insomnia often go hand in hand [128]. Both are frequently treated with sedatives. In 1991, around 4% of Americans were taking prescribed hypnotic sleep aids [128], drugs that, alongside a wealth of unpleasant and unhealthy side effects, can be habit-forming and cause disturbed sleep patterns [129]. One in six adults with a diagnosed sleep disorder and one in eight adults with trouble sleeping use pharmaceutical sedative and hypnotic medications [130].

Low arousal threshold represents perhaps the greatest risk for OSAHS patients of all the phenotypes. The risk of all-cause mortality is inversely proportional to the duration of apneic events [56]. Butler et al. studied 5712 men and women with sleep apnea. A total of 1290 deaths occurred over the 11-year follow-up. After adjusting for demographic factors (a mean age of 63 years, a mean AHI of 13.8 (standard deviation 15.0) smoking and cardiometabolic disease), it was observed that individuals with the shortest apneic events had a “significant hazard ratio for all-cause mortality”. This relationship was seen in both men and women and was strongest in patients with moderate sleep apnea [56]. The short duration of respiratory events, which is a marker of low arousal threshold, predicts mortality. It is important to perhaps state the obvious that the reason apneic events are shorter is because the individual wakes up.

Slow, nasal breathing activates the parasympathetic nervous system via the vagus nerve [131]. Mouth breathing involves fast, upper chest breathing, which is associated with sympathetic activation [132]. BRE uses exercises to reduce the respiratory rate and activate the diaphragm in order to achieve homeostatic balance between the parasympathetic and sympathetic branches of the ANS, therefore reducing sympathetic activation. Acetylcholine, which is secreted by the vagus nerve, the main driver of the parasympathetic nervous system, is instrumental in sleep, performing functions, including the activation of neurons that induce REM muscle atonia [133]. Individuals with high anxiety and chronic stress can have difficulty falling asleep and staying asleep [133]. By practicing a breathing rate of 6 bpm, sympathetic tone is reduced, and parasympathetic tone is optimized [14]. This is also beneficial for patients with comorbid depression and sleep disorders.

**Figure 4:**
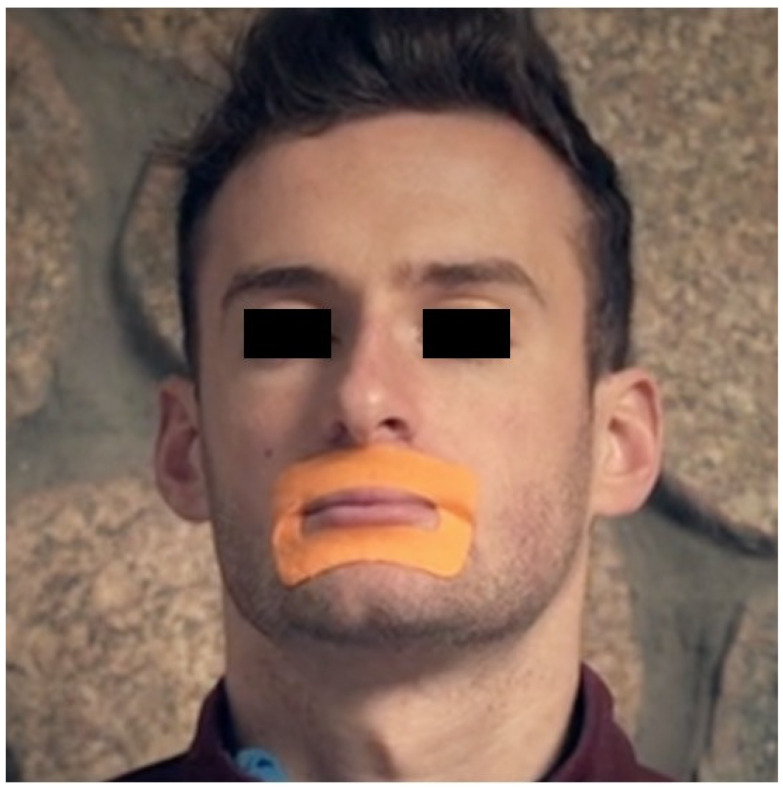
MyoTape^®^, a device to reinforce nasal breathing. A video demonstration is as Supplementary Materials (Video S2).

BRE involves re-establishing nasal breathing during rest, exercise and sleep. This includes the practice of taping the mouth during sleep to ensure nasal breathing. To date, only one pilot study exists to confirm the effectiveness of mouth taping. Thirty patients with an AHI of between five and 15 events per hour slept with their mouth closed using a porous oral patch (POP). The median AHI score was significantly decreased by using a POP from 12.0 per hour before treatment to 7.8 per hour during treatment [134]. Taping raises patient concerns that covering the mouth during sleep may be unsafe [135]. One recent product on the market, MyoTape^®^, does not cover the mouth. Instead, it surrounds the mouth, bringing the lips together with light elastic tension to help ensure nasal breathing. If at any time, the user needs to open their mouth, they can do so easily (Figure 4).

## 3. Discussion

It is important to examine OSAHS with an awareness of all four phenotypes/endotypes. This is not just an anatomical issue. Even in terms of Pcrit, the speed and volume of airflow are as relevant as the collapsibility of the airway. It is necessary to open the airway in order to help the anatomy. Equally, it is necessary to reduce breathing rate and flow to minimize airway turbulence during sleep [136], as proposed by Birch [137,138].

An interesting point to note in terms of research history is that when Evans and Lum began examining hyperventilation syndrome in the mid-1970s, their work roused considerable resistance and even hostility. It was suggested that they had misdiagnosed asthma, allergies and “non-disease” [139]. This underlines the need to keep an open mind. It is necessary, for instance, for the otolaryngologist to rule out hyperventilation syndrome when they attend patients with prior nose surgery with normal anatomic findings and subjective unsatisfactory functional results.

**Figure 5:**
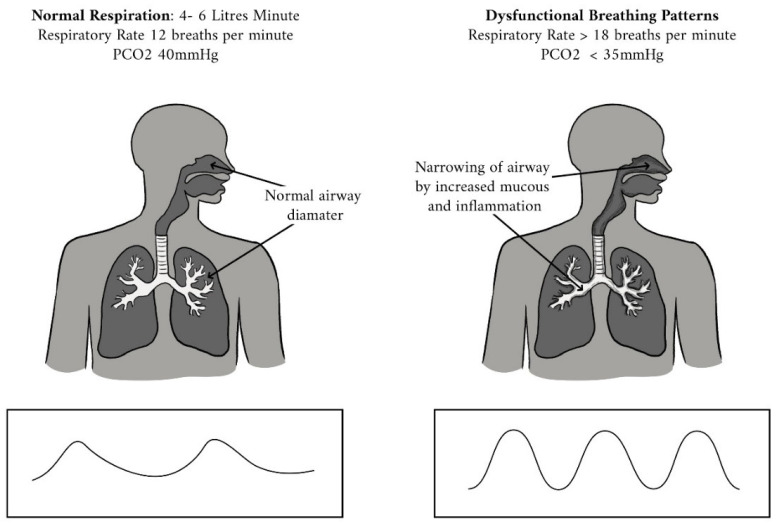
Normal breathing versus hyperventilation. Modified from www.RespiracionNormal.org [154].

Hyperventilation is now known to contribute to conditions including anxiety disorder and asthma [140,141,142,143]. Patients with hyperventilation and breathing pattern disorders demonstrate chronic abnormalities in breathing control and increased responses to CO_2_ (Figure 5) [144,145]. Hyperventilation is also associated with both weakness and hyperactivity of the breathing muscles [76,146,147,148,149]. This abnormality occurs as the primary or major contributing diagnosis in as many as 10% of all general medical patients and up to 25% of all patients complaining primarily of “dizziness” or “fainting” [150,151]. Hyperventilation syndrome has been associated with empty nose syndrome (ENS) in more than 70% of patients diagnosed with ENS. Before nasal surgery is proposed, patients should be encouraged to improve their nasal breathing, avoiding so-called “nasal underuse syndrome” (NUS) [152,153].

Chronic behavioral hyperventilation has also recently been identified in the pathophysiology of OSAHS, central apnea and mixed apnea [4]. Practicing reduced volume breathing to raise CO_2_ levels during wakefulness could impact the chemoreceptor response to CO_2_ during sleep. Current treatment protocols center on the administration of CO_2_ after an apnea [155]. However, repeated exposure to intermittent hypoxia/hypercapnia on a daily basis over the course of ten days resulted in a decrease in AHI scores in patients with OSAHS [156]. Some studies have also reported improved oxygen levels after breathing training [86,157]. However, this is not an overnight fix. Those BRE protocols that have successfully raised resting CO_2_ tended to be intensive and long-term [75,77,158,159,160,161].

There is a role for BRE and MT in SDB, both as a support for existing treatments including CPAP and MAD and for individuals with poor CPAP compliance or who fail to respond to MAD. The foundation of BRE is full-time nasal breathing. This alone can make a significant difference in the severity of sleep apnea. In order to ensure nasal breathing during sleep, props such as MyoTape^®^ provide an essential aid when it comes to re-educating the body and addressing the habitual nature of mouth breathing. With nasal breathing comes correct tongue resting posture. The tongue cannot rest on the roof of the mouth when the mouth is open. Mouth breathers keep their tongue in a lowered position [160], and habitual mouth breathing is often accompanied by a habitual tongue thrust [77].

Restoration of diaphragm function helps support lung volume and protects against airway collapse. In terms of loop gain, chemosensitivity to CO_2_ can be lowered and BHT increased. Arousal of the sympathetic nervous system can be lessened.

OSAHS is a serious condition that greatly impacts quality of life. Nasal breathing during sleep has been found beneficial in improving quality of life in SDB [75]. Existing treatment options for OSAHS are limited, cause side effects and can be subject to non-compliance. More to the point, they fail to accommodate the fact that four distinct phenotypes of OSAHS exist.

## 4. Conclusions

More research is urgently needed to investigate the therapeutic benefits of restoring nasal breathing and functional breathing patterns across all three dimensions (biomechanical, biochemical and resonant frequency). This involves:

- Nasal breathing during rest and sleep.
- Practicing reduced breathing volume during wakefulness to expose the body to slightly elevated carbon dioxide in order to reduce the chemosensitivity to CO_2_.
- Low breathing with greater amplitudes of the diaphragm and improved respiratory muscle strength.

For individuals with sleep apnea, the goal should be to reach a comfortable breath-hold time after an exhalation of 25 s. While mouth taping is effective, merely taping the mouth during sleep is not enough. Nor is it sufficient to target only one dimension of breathing. BRE needs a tailored approach to the individual. Managed in this way, it could offer substantial therapeutic intervention across all four phenotypes of sleep apnea. It would seem much of the groundwork has been done. It is time to follow the research to its logical conclusion.

## Figures and Tables

**Table 1:** Functional breathing and phenotypes of OSAHS: different hypotheses (UA = upper airway).

	Functional Breathing	Phenotypes of Sleep Apnea
**Nasal Breathing** (wakefulness and sleep)	Allows correct resting tongue posture Lower prevalence of lateral pharyngeal wall collapse [69] Reduces resistance to breathing during sleep [72] Improves biochemical dimensions of breathing [73] Improves biomechanical dimensions of breathing [73,74] Harnesses nasal nitric oxide during sleep [13]	Reduces high Pcrit Reduces high loop gain Improves UA recruitment Improves arousal threshold
**Biochemical**	Reduces chemosensitivity to carbon dioxide [11] Normalizes respiratory rate and tidal volume [75] Reduces negative suction pressure during inspiration Improves activity of UA dilator muscles [49]	Reduces high loop gain [5] Improves UA recruitment [43] Reduces AHI [76]
**Biomechanical**	Increases lung volume resulting in stiffening and dilation of the pharyngeal airway [44] Increases stores of carbon dioxide and oxygen [49]	Reduces high Pcrit Improves arousal threshold
**Resonance Frequency**	Improves baroreflex function [14] Increases heart rate variability [14] Increases blood gas exchange [14] Reduces chemosensitivity to carbon dioxide [77] Improves sympathovagal balance [14]	Reduces high loop gain Improves arousal threshold

## Data Availability

Not applicable.

## Supplementary Materials

The following are available online at https://www.mdpi.com/2077-0383/10/3/471/s1, Video S1: Breathing Re-Education: A video demonstration of decongesting nose exercise. Video S2: MyoTape^®^. A video demonstration of this device, designed to reinforce nasal breathing.
